# Duration of hospitalization increases the risk for long-term carriage of linezolid-resistant enterococci in critically ill patients

**DOI:** 10.1186/s13756-025-01551-4

**Published:** 2025-04-29

**Authors:** Vera Rauschenberger, Heike Claus, Charlotte Polzin, Vera Blaschke, Stefanie Kampmeier

**Affiliations:** 1 Institute for Hygiene and Microbiology, University of Würzburg, Würzburg, 97080 Germany; 2 Infection Control and Antimicrobial Stewardship Unit, University Hospital Würzburg, Würzburg, 97080 Germany

**Keywords:** Linezolid-resistant enterococci, LRE, Persistence, Risk stratification, Infection control, Antimicrobial stewardship

## Abstract

**Background:**

Enterococci are gut commensal microorganisms, which can however cause life-threatening infections especially in patients suffering from intestinal barrier disorders. Treatment of these enterococcal infections is challenging due to a variety of intrinsic and acquired antibiotic resistances. In this context, linezolid is applied as last-resort antibiotic. Our study aimed at determining linezolid-resistant enterococci (LRE) long-term carriage (≥ 10 weeks), since this is a risk factor for the development of LRE infection.

**Methods:**

In a one-year cohort study, all patients on hemato-oncology, intensive and intermediate care units were screened for LRE. To determine the molecular epidemiology, all detected LRE isolates were subjected to whole genome sequencing-based typing to investigate whether *in-host* selection or pathogen transmission was causative for LRE occurrence. Clinical and demographic data were recorded to identify risk factors for LRE clearance and persistence.

**Results:**

Long-term LRE carriage was identified in 7 of 46 (15%) patients. Duration of hospitalization differed significantly between LRE persistence (mean: 110 days; range 28–225 days) and clearance group (mean: 53 days; range 5–213 days). LRE strains mostly exhibited a high genetic core genome diversity, indicating that transmission events played a minor role.

**Conclusions:**

Our study shows that the duration of hospitalization increases the risk for long-term carriage of LRE. In contrast to other multi drug resistant bacteria, LRE carriage was rarely caused by transmission events. Thus, future infection prevention measures should focus on antimicrobial stewardship approaches next to classical hygiene strategies.

## Background

For several years, enterococci are of high importance with respect to healthcare-associated infections. Especially multi drug-resistant (MDR) enterococci, namely vancomycin-resistant enterococci (VRE), linezolid-resistant enterococci (LRE) or enterococci harbouring both resistances (LVRE) have been increasingly detected in hospital inpatients [1–3]. As linezolid is mostly used for last-resort treatment of VRE infections, rising linezolid resistances in enterococci are alarming [4, 5]. LRE colonization often precedes and thus represents a risk factor for a subsequent infection. Hence, long-term carriage of LRE might be of importance to predict an upcoming infection and to apply adequate infection prevention measures. Several studies have already assessed patients at risk for LRE colonization or infection. During outbreak scenarios, multiple sources of LRE in the patient environment in combination with insufficient basic hygiene measures are responsible for LRE acquisition [6–8]. In non-cluster scenarios, demographic and risk factors come into play. Here, especially comorbidities such as malignancy, immunodeficiency, previous hospitalization and prior surgery are of relevance [9–11]. Several investigations have additionally demonstrated the relationship between antibiotic use and the increasing incidence of LRE infections [4, 12–14]. However, clinical and demographic factors favouring LRE persistence or long-term colonization as a per se risk factor for LRE infection are lacking. As enhanced antibiotic prescriptions and intensified hygienic efforts are in particular needed in critically ill patients, it is often challenging to differentiate whether LRE colonization occurs due to pathogen transmission or *in-host* selection.

Aims of this study were therefore (i) to identify the proportion of long-term LRE carriage and associated risk factors favouring LRE persistence in a cohort of critically ill patients and (ii) to elucidate the way of LRE emergence using whole genome sequencing-based typing.

## Methods

### Study setting

The 1438-bed University Hospital Würzburg (UHW) is a tertiary care center admitting approximately 60,000 patients each year. Prior to the study period, routine screening was performed for methicillin resistant *Staphylococcus aureus*, multidrug-resistant Gram-negative bacteria and VRE according to the national guidelines [15–17]. Facing upcoming linezolid resistances in enterococci, a targeted LRE screening was implemented on a regular basis in parallel with the VRE screening of patients from hemato-oncology, intermediate care and intensive care units. For evaluating LRE prevalence, risk factors for LRE persistence and molecular epidemiology of LRE, this cohort study was conducted in 2020. Due to the retrospective nature of the present study, informed consent was not required. The present study was approved by the Institutional Review Board of the University of Würzburg (approval number: 20231107 02).

### Detection of LRE carriers

During a one-year study period, LRE positive patients with a follow-up for ≥ 10 weeks were recruited at the UHW and categorized regarding their LRE carrier status. In accordance with previously published studies regarding enterococci persistence [18], LRE long-term carriers were defined as patients with subsequent LRE detection ≥ 10 weeks after initial positive test.

### Microbiological culturing, antibiotic susceptibility testing

Patients were screened for LRE by rectal swab sampling on admission, weekly and upon discharge. The swabs were used to inoculate an enrichment broth based on BBL Enterococcosel broth (Becton Dickinson, Franklin Lakes, NJ, USA) supplemented with 3 mg/L linezolid (Sigma-Aldrich, St. Louis, MO, USA). After blackening, the broth was subcultured on BBL Enterococcosel agar (Becton Dickinson) supplemented with 4 mg/L linezolid for up to 72 h. Species of suspicious colonies were identified by Vitek®MS (bioMérieux, Marcy l'Etoile, France). To verify screening agar results, *Enterococcus faecium* (Efm) and *Enterococcus faecalis* (Efs) underwent susceptibility testing by Vitek®2 (bioMérieux) and gradient agar diffusion (Liofilchem, Roseto degli Abruzzi, Italy) in accordance with the current European Committee on Antimicrobial Susceptibility Testing (EUCAST) standards for clinical breakpoints [19].

### Routine infection control measures and nosocomial LRE

In case of LRE detection, patients are isolated in single rooms. Health care workers (HCW) and visitors are advised to wear personal protective equipment including gloves and gowns. Surface disinfection is performed at least once a day on a routine basis. Patient isolation can be stopped if three rectal screenings (one week apart) are negative for LRE in the absence of antibiotic treatment. LRE detections are classified as nosocomial colonisations or infections if they occur > 48 h after hospitalization and if an initial screening was negative or not performed.

### Identification of patient risk factors

Risk factors known to favour colonization with MDR enterococci and described previously [18, 20] were recorded for all LRE positive patients. In brief, these included demographic and clinical risk factors such as age, sex, duration of hospital stay, application of antibiotics, immunosuppression due to underlying diseases or treatments and the occurrence of an LRE infection.

### Whole genome sequencing-based typing

For elucidation of molecular epidemiology, isolated LRE were subjected to whole genome sequencing-based typing (WGS) using the Illumina NextSeq platform (Illumina Inc., San Diego, USA). After quality trimming and de novo assembly coding regions were compared in a gene-by-gene approach (core genome Multilocus Sequence Typing, cgMLST) using the SeqSphere^+^ software version 9.0.12 (Ridom, Münster, Germany) and the published Efm [21] or Efs cgMLST target scheme [22], respectively. To display the clonal relationship of genotypes and to differentiate between transmitted LRE and LRE selected via antibiotic application, the minimum spanning tree algorithm was applied also using SeqSphere^+^. Genotypes differing in ≤ 3 alleles (Efm) or ≤ 7 alleles (Efs) were assumed to be closely related. For backwards compatibility with classical molecular typing, the Multilocus Sequence Typing (MLST) Sequence Types (STs) and underlying linezolid resistance mechanisms were extracted from WGS data in silico.

### Statistical analysis

All data are expressed as absolute numbers or percentages, if not stated otherwise. For risk factor evaluation univariate analysis using the Fisher’s exact test was applied for categorical and the Mann–Whitney U test for not normally distributed metric data. Afterwards a multivariate logistic regression was performed, defining LRE persistence/clearance as dependent variable. Statistical significance was declared at p ≤ 0.05. All statistical analyses were performed using R Studio (R version 4.2.0) (The R Foundation, Vienna, Austria).

## Results

### Characteristics of detected LRE strains and core genome comparison

Of 3,714 patients in total, 53 LRE (46 first and 7 subsequent isolates) were isolated from patients with follow-up screening in 2020. There were no discrepancies comparing the screening agar with Vitek®2 and gradient agar tests, respectively. Of all detected LRE, 35 first and 7 subsequent isolates were of Efm and 11 first and no subsequent isolates were of Efs species (ratio Efm: Efs = 3.8:1). Of all subsequent isolates, 1 isolate harboured an additional vancomycin resistance (*vanB*), not detected in the initial isolate. While 35 first detections (all Efm) were classified as nosocomial according to above mentioned criteria, 18 cases were community-acquired. No patient with clinical signs of an infection occurred during the study period.

**Fig. 1:**
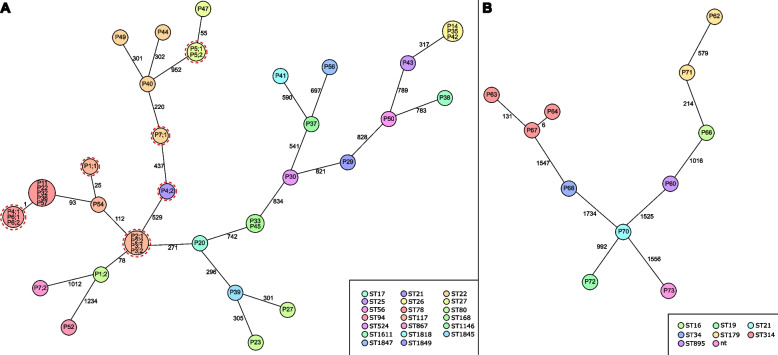
Minimum spanning tree of Linezolid-resistant (LR) *E. faecium* (**A**) and *E. faecalis* (**B**). Core genome Multilocus Sequence Typing (cgMLST) minimum spanning tree based on 1423 (**A**) and 1972 (**B**) target genes, respectively, of LR enterococci isolated from patients (P) presenting clinical LRE clearance and persistence (defined as positive subsequent screening or clinical sample, outlined in *red*). Missing values were pairwise ignored. First and subsequent isolates are shown, the latter if available (e.g. P1;1, P1;2). Size of dots correlates with the number of identical genotypes. Numbers next to connecting lines indicate numbers of alleles varying between different genotypes. Multilocus Sequencing Typing (MLST) Sequence Types (ST) extracted from sequencing data in silico are displayed by colour

Whole genome sequencing and subsequent species-specific minimum spanning tree analysis based on 1423 (Efm) and 1972 (Efs) target genes resulted in 23 singletons and 6 clusters for Efm isolates, of which 2 exclusively comprised genotypes of isolate pairs (first and subsequent isolate) of patients P2, P3 and P5 (Fig. 1A). Another cluster harboured an isolate pair (P6) and a first isolate derived from another patient (P4), while 3 clusters consisted of first isolate genotypes of 2 (P33, P45), 3 (P14, P35, P42) and 5 patients (P11, P22, P32, P36, P57), respectively, indicating pathogen transmission. Subsequent Efm isolates harboured the same genotype with the first isolate in 4 (P2, P3, P5, P6) of 7 cases. Clustering of isolates associated with LRE persistence or clearance was not detected. Analysis of Efs isolates resulted in 11 singletons with no genetic relatedness (Fig. 1B). Predominant Multilocus Sequence Typing (MLST) Sequence Types (ST) extracted in silico were ST 78 (23%), ST 117 (17%) followed by ST 22 (11% each) and ST 168 (9%) in Efm and ST 314 (27%) and ST 179 (18%) in Efs, respectively (Fig. 1). Underlying linezolid resistance mechanisms of identified Efm and Efs are listed in Table 1. In 3 of the investigated isolates, no genotypic resistance mechanism could be observed.

**Table 1:** Linezolid resistance mechanisms detected in *E. faecium* (Efm; *n* = 42) and *E. faecalis* (Efs; *n* = 11) isolates

Species	n	optrA	poxtA	23S G2505 A	23S G2576 T
Efm	6	X	X		
	1	X			
	18		X		
	14				X
	3				
Efs	7	X			
	2				X
	1			X	
	1			X	X

### Patients’ characteristics and risk factors for LRE long-term carriage

**Fig. 2:**
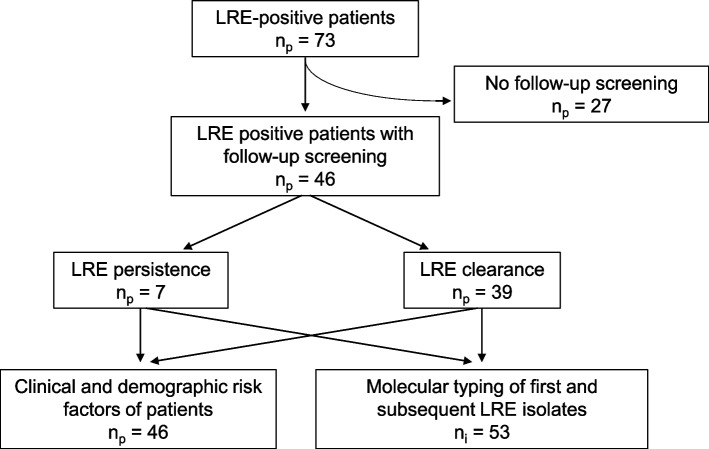
Linezolid-resistant enterococci (LRE) colonized patients meeting inclusion criteria. n_i_ and n_p_ represent the numbers of investigated isolates and patients, respectively

In total, 73 patients were identified LRE positive in 2020. Follow-up results at least 10 weeks after initial LRE detection were available for 46 patients (Fig. 2). Average age of these patients was 62 years (range 23–83 years), of whom 18 (39%) were of female sex. Demographic and clinical characteristics comparing risk factors of patients with LRE persistence and clearance are shown in Table 2. In total, 15 hemato-oncology patients received trimethoprim/sulfamethoxazole, while one patient received ciprofloxacin for antibiotic prophylaxis. Of all 46 LRE positive, two and five patients were treated with vancomycin and linezolid, respectively. No patient of the cohort received daptomycin. Univariate and multivariate statistical analysis revealed total duration of stay as independent risk factor for LRE long-term carriage (see also Table 2). Patients with LRE persistence were hospitalized twice as long as patients with LRE clearance (OR = 1.06).

**Table 2:** Comparison of clinical and demographic characteristics of patients with LRE-persistence and LRE-clearance

Characteristic	LRE-persistence	LRE-clearance	*p*-value*
Sex (male)	5 (71%)	23 (59%)	0.33
Age [years]	58 (32–77)	62 (23–83)	0.78
Oncological disease	3 (43%)	21 (54%)	0.07
Immunodeficiency	5 (71%)	31 (79%)	0.53
Antibiotic treatment	6 (86%)	29 (74%)	0.31
Liver dysfunction	3 (43%)	7 (18%)	0.18
Kidney dysfunction	5 (71%)	16 (41%)	0.20
Admission from another hospital	1 (14%)	8 (21%)	0.30
Hospitalization on an ICU	2 (28%)	15 (38%)	0.13
Duration of hospitalization [days]	110 (28–225)	53 (5–213)	**0.02**

^*^ multivariate analysis; LRE-persistence (*n* = 7) and LRE-clearance (*n* = 39)

## Discussion

In the context of an increasing burden of disease due to invasive VRE infections [23], linezolid as a last-resort antibiotic gains upcoming importance. Hence, the development of linezolid resistance poses a threat to healthcare, in particular facing the possibility of resistance transfer via a great variety of mechanisms and reservoirs [20, 24–26]. Under immunodeficiency conditions, enteric microorganisms can translocate from the gut into the blood stream or tissue (“leaky gut”) leading to an infection [27].Furthermore, persistence of LRE colonization in critically ill patients increases the chance of a LRE infection. Therefore, we here performed a study systematically assessing risk factors that favour persistence of LRE colonization, especially concentrating on patients suffering from oncologic disorders or being admitted to intensive or intermediate care wards.

During a one-year screening period, 73 LRE positive patients were detected while 4-times more patients were colonized with Efm than Efs, which is comparable to recently published data [28]. In accordance with previous data [29], a variety of underlying linezolid resistance mechanisms was identified. Among patients with a follow-up period of ≥ 10 weeks after first LRE detection only 15% were long-term carriers, whereas 60% VRE long-term carriers were identified in a previous study [18]. On the one hand, this could be a bias due to the total number of LRE detected, which is comparably low. On the other hand, this can also indicate the different mode of resistance mechanisms. In contrast to VRE for which several outbreak investigations and clonal transmission events have been described, also pointing out the outstanding role of the hospital environment as a source of VRE spread [30–32], LRE resistance rather develops with greater genetic diversity of the enterococci core genome. By comparing LRE genomes by a cgMLST approach, only 2 Efm clusters with 2 and 3 cases, respectively, could be elucidated indicating transmission events. At the same time also i*n silico* extraction of MLST ST resulted in a great diversity of LRE strains (Fig. 1).

In our statistical analysis of risk factors, duration of hospital stay significantly differed between the patients with LRE persistence and LRE clearance. Hence, duration of stay, which was also identified to be conducive in LRE colonization development [33], seems to be an independent risk factor for LRE long-term carriage in critically ill patients. This is remarkable, as application of antibiotics was also included in the multivariate logistic regression and did not result in significant differences between both groups. Nevertheless, as only a small number of transmissions was detected by comparison of core genomes, antibiotic pressure and subsequent *in-host* selection is the most feasible mode of LRE occurrence. Of note, subsequent LRE isolates of three patients were not genetically related to first isolates, indicating at least a second acquisition of LRE while treatment (Fig. 1). In the context of these results, the current recommendations for handling of patients with LRE colonization or infection, i.e. the need of intensified hygiene measures including contact precautions as reaction to outbreak scenarios [13], are questionable. Given present findings, infection prevention strategies should also focus on antimicrobial stewardship approaches to reduce the number of LRE long-term carriers. However, future studies are needed to investigate the impact of specific antibiotic substances or subclasses in increasing the risk of LRE persistence, especially as our hospital-wide antibiotic use metrics were below national benchmark data in 2020 (glycopeptides = 5.57; daptomycin = 0.1; linezolid = 1.52 DDD/patient days).

The present study comprises limitations. First, as already mentioned, the number of patients colonized with LRE was quite small resulting in possible statistical bugs. Immortal time bias may have also occurred, as during a prolonged hospital stay a chance of subsequent LRE detection will be higher compared to hospital stays of 10 weeks. Nevertheless, as screening was systematically performed in all patients at potential risks for LRE colonization, the patients and isolates evaluated are from a representative sample. Second, due to the nature of our investigation we had to exclude patients for whom follow-up screening was not available because of missing readmission or screening prior to 10 weeks after the first LRE positive test. This might lead to a potential bias in investigated demographic and clinical patient risk factors as “healthier” or “sicker” patients might have been excluded. However, neither of these limitations hindered the achievement of the study’s main objectives, namely, the determination of the LRE persistence rate and the identification of risk factors associated with LRE long-term carriage.

## Conclusions

In summary, we identified 15% LRE long-term carriers among LRE positive patients favoured by the duration of hospital stay. In the context of efficient infection prevention, transmission events play a minor role in the evaluation of LRE acquisition underlined by analysis of LRE core genomes.

## Data Availability

Whole genome sequencing data of analyzed LRE genomes were submitted to NCBI database (BioProject ID: PRJNA1097468).

## Abbreviations

cgMLST: Core genome Multilocus Sequence Typing
Efm: *Enterococcus* *faecium*
Efs: *Enterococcus* *faecalis*
LRE: Linezolid-resistant enterococci
LVRE: Linezolid- and vancomycin-resistant enterococci
MDR: Multi drug-resistant
MLST: Multilocus Sequence Typing
ST: Sequence Types
UHW: University Hospital Würzburg
VRE: Vancomycin-resistant enterococci
WGS: Whole Genome Sequencing-based typing
